# Understanding patient demand for and use of antibiotics for upper respiratory tract infection: A qualitative application of the Necessity-Concerns Framework in Saudi Arabia

**DOI:** 10.3389/fphar.2024.1399698

**Published:** 2024-06-19

**Authors:** Nouf Almeshal, Holly Foot, Amy Louise Clarke, Amy Hai Yan Chan, Rob Horne

**Affiliations:** ^1^ Centre for Behavioral Medicine, Research Department of Practice and Policy, UCL School of Pharmacy, University College London, London, United Kingdom; ^2^ Clinical Pharmacy Department, School of Pharmacy, King Saud University, Riyadh, Saudi Arabia; ^3^ School of Pharmacy, University of Queensland, Brisbane, Australia; ^4^ School of Pharmacy, Faculty of Medical and Health Sciences, University of Auckland, Auckland, New Zealand

**Keywords:** antibiotics, patient, beliefs, Necessity-Concerns Framework, upper respiratory tract infections (URTIs), antimicrobial resistance, qualitative

## Abstract

**Background:**

Reducing antimicrobial resistance (AMR) is a priority for public health. Inappropriate patient demand is an important driver of unnecessary antibiotic use. To develop an effective intervention to reduce inappropriate demand for antibiotics in upper respiratory tract infections (URTIs), it is important to identify patient perceptions that influence demand for, and appropriate use of antibiotics.

**Aim:**

To identify and describe the beliefs about antibiotics necessity and concerns that patients with URTIs have, in Riyadh, Saudi Arabia.

**Method:**

An exploratory qualitative approach was used. One-to-one, face-to-face or telephone semi-structured interviews were conducted with participants recruited using purposive sampling (based on age and gender) from primary healthcare centre in Riyadh, Saudi Arabia were conducted. Only adult patients who currently experience URTIs symptoms and agreed to participate were recruited. Recruitment for interviews continued until data saturation point was reached. The interview guide explored patients’ necessity beliefs and concerns about antibiotics, AMR perceptions, and expectations from URTIs consultation. Interview transcripts were coded using QSR NVivo 12 using framework analysis informed by the Necessity-Concerns Framework to identify key motivations driving antibiotic requests and consultations.

**Results:**

the study interviewed 32 participants (44% were male, average age was 36.84). Results identified that the patients often relate their personal need for antibiotics when encountering an URTIs symptoms to the type, severity and duration of symptoms. Patients also linked antibiotics with quicker recovery, generally expressing few concerns about antibiotics mainly because of its short duration of use. However, some conveyed their concern about frequent administration of antibiotics and effect on the body’s immune system function, which may make them more prone to infections in the future. Participants varied widely in their awareness of AMR; this was associated with many misconceptions, such as confusing AMR with antibiotics efficacy and tolerance. Interestingly, the interplay between necessity beliefs and concerns was observed to influence the decision to start and stop antibiotic, potentially impacting inappropriate antibiotic demand and unnecessary use.

**Conclusion:**

This study highlighted important beliefs and misconceptions about antibiotics and AMR in Saudi population which can be targeted in future interventions to reduce inappropriate demand for antibiotics and optimise appropriate usage.

## 1 Introduction

Antimicrobial resistance (AMR) is a global health threat that is exacerbated by the misuse and overuse of antibiotics. Almost 75% of antibiotic prescribing occurs in primary care, with upper respiratory tract infections (URTIs) being the most common reason for antibiotics prescriptions (Butler et al., 2009; Schroeck et al., 2015; Alkhaldi et al., 2021; ESPAUR, 2021). More than half of the prescriptions of antibiotics for URTIs’ are considered inappropriate (CDC, 2013; Schroeck et al., 2015; Bianco et al., 2018; Bel Haj Ali et al., 2022). The appropriateness of antibiotics prescribing for URTIs have been assessed in multiple studies. Prescriptions were found to be non-evidence-based, unjustified, and often unnecessary (Bianco et al., 2018; Alkhaldi et al., 2021; Bel Haj Ali et al., 2022; Bianco et al., 2022; Al-Baghli et al., 2023). Several factors were associated with unnecessary prescribing, including clinical factors such as type of symptoms and diagnosis, in addition to non-clinical factors related to the system and to the patients (Rezal et al., 2015; Bianco et al., 2018; O'Connor et al., 2018; Alkhaldi et al., 2021; Al-Baghli et al., 2023). Studies have explored patient related factors that influence antibiotics prescribing practice in URTIs. The most common factor reported to impact prescribing decision was that patients wanted, expected, asked for, and/or demanded antibiotics (Coenen et al., 2006; Tillekeratne et al., 2017a; Llor et al., 2013; Wong et al., 2016; Lauridsen et al., 2017; Linder and Singer, 2003; Tonkin-Crine et al., 2011; Rezal et al., 2015; O'Connor et al., 2018).

In a systematic review of 19 observational and experimental studies, between 62% and 73% of physicians reported feeling pressured by patients to prescribe an antibiotic in primary care settings. This perception of pressure has influenced doctors to prescribe antibiotics, although almost half of antibiotics prescribed being deemed unnecessary by prescribers (Rezal et al., 2015). Although several studies have investigated public knowledge and/or perceptions about antibiotics, their use and effectiveness, only a few have looked into patient expectations and their demand for the antibiotic. Patients’ expectations and demand for antibiotics was mainly associated with symptoms experiences and inaccurate beliefs about antibiotics (Gaarslev et al., 2016; Faber et al., 2010; O'Connor et al., 2019; McNulty et al., 2013).

In Saudi Arabia, antibiotics were available to the public without a prescription until early 2018, when Saudi Arabia began reinforcing prescribing regulations and warning against dispensing antibiotics without a prescription (Alnemri et al., 2016; MOH, 2018). To the best of our knowledge, only one cross sectional study has addressed patient expectations and demand for antibiotics to treat URTIs in Saudi Arabia. The study reported that out of 400 participants, only 17.3% had pressured their treating physician into prescribing antibiotics. However, the same study reported that 45.5% of the participants used antibiotics without a prescription, and 10.8% used the medication on the advice of a pharmacist (Hajjar et al., 2017). The low percentage of patients seeking antibiotic prescription from their physician could be related to their ability to obtain these antibiotics without a prescription even though they may not need one. However, the new law restricting the public access to antibiotics is expected to boost public demand for antibiotics from their primary care prescribers when encountering URTIs. This echoes the situation in other countries where regulations are in place to limit antibiotic access, yet doctors still face patient pressure for antibiotic prescriptions. This study will be the first in Saudi Arabia to explore the perceptions in-depth of public beliefs and perceptions of antibiotics to treat URTI symptoms following the law change restricting antibiotic access without a prescription.

Understanding individual’s beliefs about antibiotics that may relate to their demand and expectations is important for informing programs and interventions aimed at promoting responsible and appropriate use of antibiotics. Research shows that engagement with medication is influenced by how individuals judges necessity beliefs and concerns about their treatment (Horne, 2017). The Necessity-Concerns Framework has been widely used to understand medication taking behaviour. The framework can help to provide an in-depth understanding of why patients with URTIs feel the need to take antibiotics, in relation to their concerns about potential negative effects of taking antibiotics as recommended.

### 1.1 The Necessity-Concerns Framework

Research conducted by Horne and colleagues showed that despite the complicated and varied nature of patients’ beliefs and perceptions about their medications, they generally fall under two primary categories: the perception of treatment necessity (i.e., personal need for the treatment) and concerns regarding potential harms or negative effects (Horne et al., 1999; Horne, 2003) Individuals’ beliefs about the need for their specific treatment and their concerns about it were found to be strongly associated with patient medication taking behaviours (Horne et al., 2013; Foot et al., 2016). The Necessity-Concerns Framework (NCF) provides a conceptual model in which patients assess their beliefs about treatment necessity along with any concerns they may have about the treatment negative consequences (Horne et al., 1999). Necessity beliefs can be thought of as the answer to two questions: “How much do I need this treatment to achieve a goal that’s important to me?” and “How much can I get away without it?”. Evaluation of treatment necessity beliefs are influenced by factors such as illness perceptions (e.g., type, severity and duration of symptoms). Other factors also contribute to the development of treatment necessity beliefs, such as past experiences, social and cultural norms, information adopted from different sources, and general beliefs about pharmaceutical medicines (Horne et al., 2019). It is essential to distinguish necessity beliefs from efficacy beliefs, as they serve different roles in the perception of treatment. Although efficacy beliefs may contribute to perceived necessity, necessity beliefs reflect a patient’s perception of the need for a specific treatment (Horne et al., 1999). Patients might understand scientific evidence validating the benefits and the effectiveness of a certain treatment, but still perceive a low necessity for the treatment. While concerns can arise from the experience of symptoms as side-effects or being concerned from medication tolerance or long-term effects of medication on the body, it could also arise from wider beliefs about medicines in general (Horne et al., 2019). These more general concerns can be related to social representation of medicine as harmful or overused. Other concerns can be related to a specific type of medicine like concerns of analgesics being ineffective when overused (Horne, 2017).

Necessity beliefs and concerns are assessed by the validated Beliefs about Medicines Questionnaire (BMQ) (Horne et al., 1999). The BMQ has a necessity subscale and concerns subscale to measure beliefs about treatment necessity and concerns about them respectively (Horne et al., 1999). Although necessity and concerns are not complete opposite, research suggest that patients go under necessity/concerns dilemma when deciding to take their treatment (Horne et al., 2013; Foot et al., 2016; Horne et al., 2019). Two meta-analyses reported a significant association between medication adherence and necessity and concerns beliefs. Adherence was associated with stronger perceptions of necessity and fewer concerns about treatment (Horne et al., 2013; Foot et al., 2016). This research suggests that patients face a necessity-concerns dilemma when they decide whether to take their treatment or not. Patients often assess the potential benefits of the treatment while considering the perceived risks and potential harm associated with it. Necessity beliefs and concerns are assessed by the validated Beliefs about Medicines Questionnaire (Horne et al., 1999; Horne et al., 2019). Although the Necessity–Concerns Framework has been widely used in understanding the impact of beliefs about treatment on medication taking behaviour in chronic conditions, only a few studies have assessed its application on medication taking behaviours during acute illnesses or in medications that are used for short period of time such as antibiotics (Chan et al., 2021; Perera et al., 2021). The studies demonstrated that the NCF has the potential to address misplaced beliefs about antibiotics and addressing misplaced beliefs about antibiotics has a successful impact on decreasing antibiotic expectations (Perera et al., 2021). However, the first study was conducted online via a paid survey platform which may produce a biased sample and the second study focused on exploring patient expectations rather than exploring the key beliefs underlying antibiotic use in people with cold and flu symptoms.

Due to the transient and diverse nature of URTIs, it is crucial to evaluate patients’ beliefs about antibiotics and about their symptoms while they are experiencing them to inform future interventions to reduce unnecessary antibiotic use for URTIs. This real-time evaluation of their perceptions is essential to capture attitudes towards URTIs and their beliefs about antibiotics and helps to minimize recall and representation biases. Therefore, this study aimed to understand how patients with URTIs judge their personal need for antibiotic and what concerns are salient to them considering perceptions of antimicrobial resistance (AMR) in Saudi Arabia.

## 2 Materials and methods

### 2.1 Design

This is an exploratory qualitative cross-sectional study that used face-to-face and telephone semi-structured interviews. The study design was carried out in line with the consolidated criteria for reporting qualitative research (COREQ) guidelines (Tong et al., 2007). One-to-one interviews were deemed most appropriate to capture participants individual experiences and beliefs about antibiotics use in URTIs. The study has been approved by UCL ethics committee (16,543/001) and an additional local ethics approval was obtained from the Ministry of Health of Saudi Arabia’s Institutional Review Board (IRB Log No. 20-014E).

### 2.2 Study sample and sample size

Purposive sampling with maximum variation (on the basis of age and gender) was used to capture all the common and unique characteristics, factors and thoughts to ensure a representative sample to achieve the study aims (Palinkas et al., 2015). The study participants’ inclusion and exclusion criteria: Adult patients aged 18–70 years old, experienced symptoms of upper respiratory tract infection (flu-like symptoms) at the time of the study and understood and spoke Arabic. Participants who were deemed too ill to participate were excluded. Participants continued to be recruited for interviews until data saturation point was reached (no novel information or themes were being generated from the interviews, and further sampling was not needed (Saunders et al., 2018).

### 2.3 Recruitment

Interviews took place on January–February 2020 in two PHC that were nominated by the MOH in two distinctive areas (north and east) in Riyadh, Saudi Arabia. Potential participants who presented to the primary healthcare centre (PHC) with URTIs symptoms were identified and approached by the site nurse who provided them with the study participant information sheet and notified the researcher accordingly. Participants who showed an interest in taking part in the study were invited to a consultation room at the PHC for a face-to face interview or telephone interview. Consent forms were provided by the researcher afterwards and consent was obtained from participants prior to interviews. Most interviews were conducted after the clinic consultations. However, some patients requested their interviews to be conducted while they were waiting for their consultation appointment.

### 2.4 Interview

One-to-one semi-structured interviews were conducted by the lead author (NA). Interviews were audio-recorded, and followed a standardized topic discussion guide, informed by the literature and the Necessity-Concerns Framework (Tillekeratne et al., 2017b; Hawking et al., 2017; Horne et al., 2019; Chan et al., 2021). Hence the Necessity-Concerns Framework is operationalised using the validated Beliefs about Medicine Questionnaire (BMQ), and the BMQ was used to assist in formulating the interview questions (Horne et al., 1999; Chan et al., 2021). A detailed discussion guide with all questions is shown in supplement (1).

The topic guide was piloted in one-to-one interviews with five healthy adult participants, two in English, to ensure the appropriateness of the questions. It was then translated using forward and backward translation into Arabic. The Arabic translation was further piloted in another three one-to-one interviews with Arabic-speaking healthy individuals to ensure the clarity and the appropriateness of the questions. Participants were offered incentives for their time and participation upon completion of the interviews with a SAR 100 (£20) voucher.

### 2.5 Analysis

Interviews were transcribed verbatim in the Arabic language; transcripts were then translated into English aided by a professional academic translator. The English transcripts were further checked against the original Arabic transcripts by the bilingual researcher (NA) to verify accuracy of transcriptions and translation (Naqvi et al., 2019). The English transcripts were moved to NVivo 12 pro software for analysis. Data were analysed using a framework analysis informed by the Necessity-Concerns Framework (Horne et al., 1999).

A hybrid approach of deductive and inductive coding was used (Fereday and Muir-Cochrane, 2006; Gale et al., 2013; Mortazhejri et al., 2020). During the process of transcripts coding, the deductive coding was based on the Necessity-Concerns Framework, while inductive codes were assigned to segments of transcripts’ data that describes potential new themes and seemed relevant to the original research question, or impacted participants necessity beliefs and/or concerns about antibiotics, such as factors related to the context of Saudi Arabia.

To ensure reliability of the coding, 25% of transcripts were anonymously and independently analysed by two researchers (NA, ALC). All themes identified during the analysis were discussed between the research team and discrepancies related to the themes were dealt with through consensus. The lead author is a qualified clinical pharmacist with training and experience in research and have received training in conducting interviews and in qualitative assessment and analysis. All members of the research team have extensive experience in thematic analysis and behavioural medicine.

## 3 Results

In total, 34 individuals were approached, and 32 participants agreed to participate and were interviewed from two PHC in Riyadh, Saudi Arabia. In this sample, 31% of participants were aged 18–29, 28% were aged 30–39, 28% were aged 40–49, and the remaining 13% were 50 years and older. The average age in this sample was 36.84 (Age ranged between 20–62 year-old), and 15 (47%) participants were male. A good variation of educational level was also observed: 6% of the participants held a higher education degree, 56% had a university degree, 32% had a secondary school certificate, and only 6% had an intermediate school education (refer to Table 1 for participants characteristics). Overall, 11 themes were identified, nine of these themes were related to the content of Necessity and Concerns beliefs: three themes under necessity beliefs, five themes related to treatment concerns, and one theme described the interplay between necessity and concerns. In addition to the content of necessity beliefs and the content of concerns, the study highlighted themes related to the context which Included two themes. A summary of themes and subthemes are available in Table 2.

**TABLE 1:** Summery of participants.

Participant number	Age	Gender	Education	Symptoms	Interview mood
1	21	Female	University	Runny nose, dry chesty cough, fever, sore throat	Face to face
2	30	Male	University	Runny nose, chesty cough, sore throat, sneezing	Face to face
3	39	Female	University	Fever, sore throat, lethargy, headache	Face to face
4	41	Female	University	Dry cough, fever	Face to face
5	46	Female	University	Runny nose, dry cough, sore throat, sneezing	Face to face
6	21	Male	University	Chesty productive cough, sore throat, fever, sinus pain	Face to face
7	37	Male	Higher education	Runny nose, chesty cough, sneezing	Face to face
8	34	female	Higher education	Runny nose, dry chesty cough, sore throat	Face to face
9	56	Male	University	Runny nose, sore throat, fever	Face to face
10	23	Male	University	chesty cough	Face to face
11	32	Female	University	Runny nose, dry cough, sore throat, sneezing	Telephone
12	39	Female	Secondary school	Runny nose, productive cough, sore throat, fever	Face to face
13	25	Male	University	Chesty cough, fever, sore throat	Face to face
14	20	Female	University	Runny nose, dry chesty cough, fever, sore throat, sneezing	Face to face
15	33	Male	University	Runny nose, chesty cough, sore throat, sneezing	Face to face
16	41	Female	Intermediate school	Runny nose, dry chesty cough, fever, sore throat	Face to face
17	63	male	University	Chesty cough, sore throat, Asthma	Face to face
18	46	male	Secondary school	Chesty cough	Face to face
19	39	male	Secondary school	Runny nose, chesty cough, sore throat, sneezing	Face to face
20	54	Female	Intermediate school	Runny nose, chesty cough, productive cough, sore throat, sneezing	Face to face
21	20	male	Secondary school	Chesty cough, fever, sore throat	Face to face
22	26	Female	University	Dry cough, fever, sore throat	Face to face
23	47	Female	Secondary school	Runny nose, dry cough, sore throat, sneezing	Face to face
24	37	Female	Secondary school	Runny nose, sore throat	Face to face
25	26	Male	University	Chesty cough, sore throat, fever, sneezing	Face to face
26	44	Male	Secondary school	Chesty cough, sore throat, lethargy, shortness of breath	Face to face
27	29	Male	University	Runny nose, sore throat	Face to face
28	45	Male	Secondary school	Runny nose, sore throat, sneezing, enlarged tonsils, cold, headache	Face to face
29	52	Female	Secondary school	Sore throat, tonsilitis	Face to face
30	45	Female	University	Sore throat	Face to face
31	42	Female	Secondary school	Runny nose, productive cough, sore throat, fever, loss of voice	Face to face
32	26	Female	University	Runny nose, chesty cough, productive cough, sneezing	Face to face

**TABLE 2:** Themes.

Content themes	Necessity beliefs	• N1: Impact of illness perception on antibiotics necessity • N2: Preference of antibiotics and quick recovery • N3: Antibiotics is the only solution
	Concerns	• C1: Low concerns • C2: Concerns about allergic reaction and SE • C3: Impact on immunity, and frequent use of antibiotics • C4: Effective antibiotics perceived to be more harmful • C5: Low concerns about antimicrobial resistance
	• interplay between necessity and concerns and the decision to start/stop antibiotic	
Context Themes	• T1: Role of the doctor in managing beliefs • T2: Impressions on policy changes	

### 3.1 Themes related to necessity beliefs

#### 3.1.1 Theme N1: Impact of illness perception on antibiotics necessity

Participants described how their perceived need for antibiotics was influenced by lay diagnosis, symptoms type (e.g., fever, phlegm, extreme lethargy), symptoms severity, and duration of symptoms.

“When my throat is very congested and sore and I have tonsillitis in addition to having terrible headache, I feel I need them. I may also have high temperature and a decrease in blood pressure” (Participant 29, F, 52).

“If I feel I am suffering from a disease, such as congestion or others, in case I keep having the symptoms for more than three days and they do not get better, I feel that I need antibiotic.” (Participant 25, M, 26).

“Maybe if my disease last longer and my state deteriorate more and more. In these cases, I feel I must take [antibiotic] ………in addition to temperature….” (Participant 05, F, 46).

However, participants in our sample showed variation in their attitude toward URTIs. Some participants had a low threshold for seeking help and chose to visit the clinic upon symptom onset for appropriate treatment. While others reported managing URTIs by initially using alternative remedies or over-the-counter (OTC) medications, and wait for symptoms to deteriorate before visiting the clinic, which may implicitly contribute to perceived need for antibiotic:

“[I wait] till symptoms become more severe; for one or two days…… until the situation gets worse.” (Participant 03, F, 39).

“I usually do not like to wait for the last stage when I become very sick. When someone first feels sick, he should go right away to a hospital because symptoms are mild at this early stage.” (Participant 09, M, 56),

“I have warm drinks, take Panadol, cold and flu and gargle. But if I have a fever and it lasted for two or three days, I would resort to taking an antibiotic” (Participant 30, F, 45)

“In case my fever lasted for more than two days, I would visit the hospital or clinic and start taking medication.” (Participant 11, F, 32).

In addition, the perceived worsening of symptoms or symptoms deterioration was often linked to the sense of need for antibiotics. However, the slow, or weak impact of alternative remedies or OTC might have led participants to perceive their symptoms as worsening or not improving, thereby reinforcing their perceived need for antibiotics.

“I have been having hot drinks for 3 days, applying Vicks and then taking paracetamol with no improvement. I still feel the phlegm coming and chest infection …….. the treatment that she gave me never benefitted me. Then, I would necessarily take the antibiotic, definitely” (Participant 12, F, 39).

“Because I feel that I currently need the antibiotic, it has been 4 days or 5 days now and I am having the same symptoms. If I feel that every day I am worse than the day before or I do not get better or I get much worse, here I feel I have to take an antibiotic……because I feel the symptoms remain the same even after taking some medications; they did not improve” (Participant 25, M, 26).

#### 3.1.2 Theme N2: Preference of antibiotics and quick recovery

Some participants referred to antibiotics as a desirable thing even if they would not explicitly request it from their prescribers. They also expressed their preference for antibiotics because they believed it can fasten the recovery process, or prevent worsening of the symptoms.

“antibiotic speeds up the process of the recovery; meaning it [antibiotic] does not harm the body.” (Participant 04, F, 41).

“I feel it’s the quick solution” (Participant 24, F, 37).

“I should start taking treatments before the symptoms get worse as I have a constant fever” (Participant 11, F, 32).

Participants also referred to the benefit of quicker recovery to achieve goals that are important to individuals, and these goals will vary from being able to perform usual activity to being able to attend an important event, or trip.

“having an important wedding occasion, a special occasion or sometimes very important business meetings, I would have to take one so as to recover quickly before two or three days in which I could not recover by visiting a doctor and taking other treatments” (Participant 15, M, 33).

“as a mother, I have to do everything. It’s difficult. The effect of warm drinks is slow, …… You know the lady of the house must be responsible, so I try to take antibiotics to help with handling the house” (Participant 24, F, 37).

“sometimes its ok to feel sick in certain days, when I have the usual fatigue, but when I feel tired like these days, when I have work, and I have to go to work **I feel I need** it this time because of my work …… It would not be as necessary during holiday or weekends” (Participant 14, F, 20).

#### 3.1.3 Theme N3: Antibiotics is the only solution

Some participants attributed their need for antibiotics to their perception of antibiotics as the only effective option to treat their symptoms. This perception became more salient after experiencing limited recovery with the use of alternative medicine or OTC treatment.

“to be honest, I feel that an antibiotic is the solution.” (Participant 24, F, 37).

“if he prescribes for me a fever reliever only or painkiller or even vitamins, the same disease will remain. I see that the antibiotic is the main cause of beating the disease.” (Participant 06, M, 21).

“When I feel desperate, I feel despaired from recovering without it, I feel I am urgently required to take an antibiotic.” (Participant 16, F, 41).

### 3.2 Themes related to antibiotics concerns

#### 3.2.1 Theme C1: Low concerns

Participants generally expressed few concerns about antibiotics mainly because of its infrequent, and short duration of use:

“No, I'm not concerned about side effects because I do not use it a lot [antibiotics] ….. ” (Participant 09, M, 56),

“I do not have a problem to take it myself” (Participant 28, M, 45).

Participants’ familiarity with antibiotics along with past positive experiences with its use have contributed to the reduced level of concern about antibiotics:

“I have never experienced any bad effect from it” (Participant 12, F, 39).

“based on my experience of taking antibiotics, because I took antibiotics before, I do not have concerns; I feel they are normal” (Participant 06, M, 21).

“No, praise be to Allah. If I am used to it and know its type, it’s Ok. If it is new, I would be more concerned, and inquire about it” (Participant 24, F, 37).

#### 3.2.2 Theme C2: Concerns about allergic reaction and side-effects

Although the sample described few concerns about antibiotics use, some participants reported being concerned about possible side effects and the possibility of developing allergic reactions to antibiotics:

“they [antibiotics] can affect the kidneys, because in many people they affected the kidneys. They also can affect badly on the stomach causing ulcers. In addition, they can cause allergic reactions in some people’s” (Participant 20, F, 54).

“it harms the kidneys and the liver. Some people who have chronic stomach pains or issues may have more complications. Maybe allergic reaction especially those who are allergic to antibiotics.” (Participant 06, M, 21).

Another participant described her concerns about developing side effects while treating her symptoms:

“I’m afraid I’m treating my present case and it is causing me a problem……… I do not know. I always feel it may affect the kidneys; it may affect any other organ in my body” (Participant 05, F, 46).

#### 3.2.3 Theme C3: Concerns about impact on immunity, and frequent use of antibiotics

Participants conveyed their concern about frequent administration of antibiotics the potential harms associated with the unnecessary use. Some participants were also concerned about the effect on the body’s immune system, which may make them more prone to infections in the future:

“using it unnecessarily can be harmful. I see that it destroys my immune system; I do not know why I have such belief” (Participant 08, F, 34).

“antibiotic can weaken the body’s immunity, so the body can no longer resist the disease” (Participant 05, F, 46).

“All I know about antibiotics is that they will make me **lose all my immunity**………… I know that the antibiotic as if to say to the body, stop, I am doing the role for you, so I did not benefit from immunity” (Participant 02, Male, Age 29).

Also, participants were worried about frequent antibiotics use and medication tolerance, or developing long-term side effects:

“harmful in a way ….. It affects the stomach and immunity is greatly weakened because the body becomes used to taking them with any illness. then the body becomes weak defending itself against other infections and requires antibiotics. It cannot resist the disease” (Participant 25, M, 26).

“frequent use causes kidney failure and liver damage ….. I mean frequently using it is not good” (Participant 23, F, 47).

#### 3.2.4 Theme C4: Perceptions of antibiotics potency, and associated harms

Antibiotics were perceived as an effective treatment. However, this belief placed a source of concern that the stronger the antibiotic, the more harmful it could be to the body. Other factors (e.g.,: the strength and size of the antibiotic pill) impacted perceptions of antibiotics’ potency and increased participants concerns.

“You know that some of these medications are a little bit strong, so I think antibiotics are among those strong ones. I feel taking more of them may lead to negative symptoms such as the stomach and others like that” (Participant 25, M, 26).

“If I had increased doses! Or if the percentage of the infection pill is higher or the proportion of the antibiotic in a medication is more. I would feel more afraid” (Participant 16, F, 41).

“When I saw the size of the pill, frankly speaking, I hesitated! I even thought about splitting it into half because I did not know what to do …….. It was big! Very big! …………….. Its large size means it is of high strength. I hesitated very much” (Participant 03, F, 39)

#### 3.2.5 Theme C5: Low concerns about antimicrobial resistance

In this sample, most of the participants were not aware about the issue of AMR. Some participants did not believe that AMR is a real public health challenge even after AMR was explained.

“I never heard about it [antimicrobial resistance] before” (Participant 10, M, 23).

“I do not know if this idea is true, or not, but I do not feel it is correct, I do not believe in it” (Participant 08, F, 34).

Although awareness of the issue of AMR was limited among participants, it was associated with misconceptions. Some participants had conflicting views regarding AMR and immunity, antibiotics efficacy, and tolerance. Some participants confused bacterial infections with viral infections, with a belief that all infections can benefit from antibiotics.

“Eventually, the body will not respond to the antibiotic with the passing of time as it will requires more and bigger doses. This is a problem in itself” *(*Participant 02, M, 31).

“It is true. If the body gets used to the antibiotic, it no longer works.” (Participant 28, M, 45).

“I hear about one of them needs an antibiotic. I hear about a type of virus or bacteria. I do not know which one of them needs an antibiotic” (Participant 05, F, 46).

### 3.3 Theme: Interplay between necessity and concerns and the decision to start/stop antibiotic

This theme describes the interaction between necessity beliefs and concerns and has been observed in the decision making process for participants when deciding to initiate or discontinue antibiotics. During the discussion. participants seemed to perceive a need for antibiotics at the onset of their illness, in contrast to their relatively low level of concerns about antibiotics when the illness was perceived as a greater threat to health:

“I say these side effects are less likely to happen compared to the possibility of worsening of the current symptoms or get a more complicated disease” (Participant 06, M, 21).

This evaluation of the need for antibiotics vs. concerns has further influenced adherence to antibiotics. Participants expressed a tendency to discontinue antibiotic usage before the recommended duration as their concerns about antibiotics became more prominent when their illness improved. Illness improvement prompted participants to reevaluate their need for antibiotics and leading them to discontinue the treatment earlier, as explained by one participant:

“Even when the doctor instructed me to take the medications for seven days; I stopped after 3 days believing I am better now and very well …………… I think this thing (antibiotic) may harm me, so why do I increase the period of taking it! Why I take more antibiotics as long as I have recovered!” (Participant 06, M, 21).

“I myself do not take more than one or two pills …….. I feel it is so strong, to keep using it frequently …. I mean … hmm, what I should tell you? it has strong effect on the body ……… I feel that I have already recovered and no longer need more of it (Participant 22, F, 26).

This interplay between antibiotics necessity and concerns has also influenced Participants’ decision to fully adhere to the antibiotic’s regimen, even when perceived as necessary.

“If the doctor prescribed an antibiotic for me and I saw that I got better after taking it for three days; I would refrain from taking it or maybe take it in fewer doses, like taking two instead of 4 pills or one instead of three pills after three days, for example, if the doctor allowed me” (Participant 19, M, 39).

“he should not continue to take a lot of them, but just one or two pills per day. If it was a strong effective kind, he should have to take one pill at morning and another pill on the next day, and only that. He should not continue to take it for long period of time of four or five days. Doctors advise to take it for just 5 days. No, I would not advise him to take for five days, but just a pill or two pills only” (Participant 16, F, 41).

“The period of using the antibiotics increase my fears in case it lasts for a long time. I feel sometimes I am compelled to take it only for three or four days at most when the doctor prescribes taking it for, for example, a week. I try to stop it on my own, especially if I feel better” (Participant 25, M, 26)

### 3.4 Contextual related themes

#### 3.4.1 Theme T1: Clinician recommendations

Participants trusted their doctor’s judgment when antibiotics were deemed not necessary and would accept their decision not to prescribe antibiotics.

“I would book an appointment and go to the doctor. I would tell him why I think I need an antibiotic. In case he agreed, he would prescribe it for me; in case he refused telling I did need it for such-and-such reasons, I would surely change my mind and not take it” (Participant 08, F, 34).

“the most important thing is the doctor’s opinion. He is the one who examines me and checks whether I need an antibiotic or not. If I do not need it, he will give me an alternative” (Participant 23, F, 47).

Some participants reported not having any concerns about taking antibiotics when prescribed by their specialized doctor. Other participants also indicated feeling secure about taking antibiotics if it was recommended by their doctor.

“when they are prescribed by a specialist and taken at the exact time and according to instructions, God willing, there is no harm in taking them” (Participant 07, M, 37).

“If the prescription is from the doctor, I will not be afraid at all” (Participant 27, M, 29).

Only few participants reported being not satisfied if antibiotics were not prescribed and would consider consulting another prescriber or private practice to obtain a prescription:

“I would visit another doctor” (Participant 11, F, 32), “[I would] go to a private practice” (Participant 24, F, 37)

#### 3.4.2 Theme T2: Impressions of the changes in antibiotic dispensing policy in Saudi Arabia

Most of the sample were supportive to the decision to ban the sale of antibiotics without a prescription, only few participants had controversial views about the policy. Those few participants preferred a more flexible access to antibiotics, particularly when they are familiar with their illness, perceiving visiting the doctor for an antibiotic’s prescription as unnecessary.

“this decision has one positive side and another negative one. For example, I am used to a certain treatment and take it and I know it. Instead of me bothering myself and disturbing the clinic for having a prescription and the like, I am able to buy it for 15 riyals (=£3)” (Patient 17, M, 63).

“it is wrong that they take antibiotics on their own, but they may need them very badly” (Patient 03, F, 39), “I think we are sometimes compelled to visit a primary healthcare centre for just having an antibiotic prescription which I really do know I need it. I sometimes know for sure what I suffer from like when I need an antibiotic cream for, for example, a wound. All of them, unfortunately, are forbidden” (patient 30, F, 45).

## 4 Discussion

This is the first study to qualitatively explore patients’ beliefs and perceptions about the use of antibiotics in managing their URTIs symptoms in Saudi Arabia. Using the Necessity-Concerns Framework, the study identified the content of participants’ antibiotic necessity beliefs and their concerns when used to treat URTIs, and how these may contribute to demand for and use of antibiotics. Other contextual factors that may impact antibiotic use were also identified.

Perceptions regarding antibiotics necessity were influenced by factors related to participants’ perception of the illness and the symptoms they experienced, the perceived effectiveness of antibiotics as the only effective treatment and in providing quick recovery accelerating the ability to perform usual activities. Personal need for antibiotics was influenced by symptom experiences with patients describing how antibiotics are more likely to be required when experiencing more severe symptoms. This finding aligns with previous literature, where antibiotics were perceived as needed based on symptom experiences, duration and deterioration of these symptoms, in addition to the successful experience with antibiotics with similar symptoms, and have contributed to expectations and demand for antibiotics (O'Connor et al., 2019; Courtenay et al., 2017; Gould et al., 2007; Mazinska et al., 2017; McNulty et al., 2013; Ong et al., 2007; Pan et al., 2016; Roberts et al., 2015; Van Driel et al., 2006). However, it contrasts with a recently published study on Canadians’ perceptions of URTIs, where most patients believed that URTI symptoms were self-limiting and would resolve without an antibiotic prescription (Mortazhejri et al., 2020). This variation could be attributed to antibiotics being restricted to prescription-only medicines. These policies might have influenced how people view the significance of acquiring antibiotics through doctor’s prescriptions, when necessary, possibly by enhancing doctor-patient interactions providing opportunities for information exchange (Mortazhejri et al., 2020; Naing et al., 2021).

The identified relationship between illness perception and people’s perception of antibiotics’ necessity beliefs can be explained by how the Necessity-Concerns Framework extends the Common-Sense Model of self-regulation. (Horne et al., 2019). The extended common-sense model helps to understand how illness representation (symptoms identity, severity, timeline, and consequences as identified in by participants in this sample) triggers the perceptions of treatment necessity (Horne, 2003; Horne et al., 2019). These illness representations (e.g.,: symptoms are severe and lasting for more than 3 days which indicate antibiotics) and inaccurate beliefs about antibiotics (antibiotics are more effective, and lack or low concerns about their use) facilitates the establishment of a common-sense fit between the URTI illness and the need for antibiotics. Taking antibiotics was identified by participants as a coping procedure which is further appraised by individuals and subjected to changes in treatment beliefs (e.g., I do not need to take the remaining of the antibiotic course because I feel better now, and it may cause side effects).

In addition, our study showed that beliefs about antibiotics necessity does not come only from the clinical experience (e.g., symptoms severity or deterioration) it can also come from other surrounding factors such as the need to return to work or attend an important event, which may increase the perceived necessity to take antibiotics. This inaccurate belief that antibiotics can be used to accelerate the recovery from URTIs symptoms or prevent their worsening have been associated with patients’ expectations and demand for antibiotics (Branthwaite and Pechere, 1996; Van Driel et al., 2006; Faber et al., 2010; Roberts et al., 2015; Gaarslev et al., 2016; Shaw Teng Pan et al., 2016; Strandberg et al., 2016; Davis et al., 2017).

In the cohort, concerns about antibiotics were primarily related to side effects, allergic reactions, and a misperceived impact on immunity. Interestingly, although some patients expressed concerns about side effects, others felt they were not a significant issue due to their previous safe experiences and the relatively short duration of antibiotics use. This lack of concern about antibiotics’ side effects has been previously reported in the literature where participants did not believe that the potential for adverse drug events was a significant issue (Roberts et al., 2015). The low levels of concerns about antibiotics use is strongly linked to expectations and demand for antibiotics (Macnamara et al., 2000; Van Driel et al., 2006; Gaarslev et al., 2016; Shaw Teng Pan et al., 2016) and this was further confirmed in our cohort.

Furthermore, participants reported being more concerned about antibiotics that are perceived to be more powerful, perceiving stronger and powerful antibiotics to be more harmful. This perception of antibiotics being a double-edged sword aligns with broader beliefs about medicine, where people often associate efficacy with toxicity, believing that effective treatments could potentially have more harmful side effects (Horne, 2003; Horne et al., 2019). This perception also contributes to necessity-concerns dilemma and was observed to influence engagement with antibiotics in this sample (Horne et al., 2019). Patients mentioned that they would discontinue antibiotics once they start feeling better to minimize potential risks. This has an important implication in understanding adherence behaviour to antibiotics, where nonadherence made common sense to patients as they believed antibiotics were unnecessary once they felt better, considering the potential risks of continued antibiotics use.

Although the study has focused on identifying beliefs that influenced patients demand and expectations for antibiotics, it identified beliefs that also influenced antibiotics overuse and misuse (e.g., not completing antibiotics course). Most importantly, the balance between beliefs about antibiotics necessity and concerns and its impact on decision making regarding antibiotics. Addressing these beliefs and misperceptions regarding URTIs and antibiotics, presents an opportunity to promoting prudent use of antibiotics, curbing inappropriate demand and, consequently, antimicrobial resistance (AMR).

Misconceptions and lack of awareness about AMR that were identified in this study are common and align with the literature (e.g., McCullough et al., 2016; Bakhit et al., 2019; Bianco et al., 2021; Licata et al., 2021; Cantarero-Arevalo et al., 2022; McNulty et al., 2022; Hika et al., 2022). A common misconception that was identified in this study and consistent with previous literature is related to the in accurate belief that all infections (both viral and bacterial) can benefit from antibiotics use (Bianco et al., 2021; Licata et al., 2021; McNulty et al., 2022).

Similar to Australian and Russian patients’ beliefs about AMR, participants in this study showed little awareness that inappropriate use of antibiotics can cause antimicrobial resistance (Bakhit et al., 2019; Cantarero-Arevalo et al., 2022). These misconceptions were also evident among certain ethnic minorities in the United Kingdom (McNulty et al., 2022). However, some participants in our study were able to articulate this link during discussion.

These common misconceptions and lack of awareness about AMR in the study sample suggests that perceptions of AMR did not seem to impact patient necessity beliefs, concerns, or expectations for antimicrobials prescription as participants never mentioned AMR unless asked about it. Although this could have contributed to the low concerns about antibiotic use that was observed in the study sample, prior studies indicated that information population-level side effects were less salient to individuals than personal-level adverse effects of antibiotics (Mehrotra and Linder, 2016; Perera et al., 2021). Previous studies have also established the need to incorporate information that addresses misconceptions about antibiotics and AMR and highlighted these misconceptions as a significant predictors of antibiotics misuse (Bianco et al., 2021; Licata et al., 2021; McNulty et al., 2022). However, incorporating information about AMR in intervention should be considered carefully and should be further tested in Saudi Arbia context as previous experimental research did not include such information in their interventions (Perera et al., 2021).

In our sample, participants explicitly mentioned that they would trust doctors’ decisions not to prescribe antibiotics and that doctors could help them overcome their concerns. It has been reported that physicians within outpatient settings responded to patient demand and prescribed antibiotics (Rezal et al., 2015). Physicians also perceived changing patient beliefs and expectations about antibiotics as time-consuming and unsatisfactory during patient consultations (Rezal et al., 2015). Therefore, we believe that improved communication between doctors and patients could lead to better antibiotics use, including reduced demand and enhanced adherence. This has further implication in designing future interventions that could have prescribers’ involvement in their designs. It is important to emphasize at this point the crucial role that community pharmacists should play in communicating information about self-medication with antibiotics. Previous studies have shown that despite legal restrictions on selling antibiotics without a prescription, pharmacists often dispensed them to patients (Bianco et al., 2021; Qudah et al., 2024).

Furthermore, the beliefs and misconceptions reported in this study serves as a foundation for developing interventions based on the well-known and widely used health psychology theory the Necessity-Concerns Framework. The NCF have proven effective in the design and implementation of interventions across various diseases and health systems to optimize medication adherence. Addressing inaccurate beliefs about the necessity of antibiotics and concerns related to their use, along with misconception associated with AMR can inform the development of tailored interventions. Previous interventions have also utilised a tailored approach in responding to specific patients’ beliefs about antibiotics necessity and concerns using online algorithms (Chan et al., 2021). Implementing structured interventions based on the NCF can serve as a valuable tool for effectively communicating information about antibiotics to the public, addressing concerns and beliefs regarding the necessity of antibiotics for treating URTIs. Establishing reliable methods to effectively communicate evidence-based information about the benefits and risks of antibiotic use and misuse to patients, at both personal and population levels, is crucial. Presenting this information in easily understandable and accessible ways can enhance awareness of antibiotic use, consequences of misuse (including self-medication, and non-adherence), and AMR. The availability of inaccurate sources of information, such as in the internet, have been identified as predictors of antibiotics self-medication with antibiotics (Licata et al., 2021). The findings of this study hold further significance for policymakers in countries where antibiotics are accessible without a prescription, contributing to a higher possibility of overuse and misuse of antibiotics. This problem of uncontrolled access to antibiotics is prevalent around the world; it has been reported across 24 countries (Auta et al., 2019). The paper provided patients perspective about antibiotics use and determinants of demand for antibiotics which is an important issue that should be considered by policymakers. Although changing policies is a crucial step toward controlling unnecessary antibiotic use, implementing policies should go side by side with delivering scientific evidence that justifies these policies to individuals using appropriate methods. This is particularly relevant for countries in the process of transitioning towards implementing regulations to control antimicrobial dispensing.

### 4.1 Strengths and limitations

To our knowledge, this is the first qualitative descriptive study to apply a theory-based approach to understand beliefs about antibiotics and AMR in the Saudi Arabia context. Furthermore, although the NCF has been widely used to understand patients’ beliefs about their chronic medication, this is the first qualitative study to use the NCF in understanding patients’ beliefs about antibiotics to treat URTIs.

Although participants bias and recall bias cannot be ruled out, the study have recruited participants with active URTIs symptoms and identified their beliefs about antibiotics while experiencing the symptoms. This is particularly important as interviewing participants with current symptoms would potentially reduce the risk of self-selection bias, and thereby providing a more representative sample for study. Also, while offering incentives can potentially introduce participation bias and influence individuals’ participation decisions, this was taken into consideration in this study. Participants were informed that they would receive incentives after completing the study interview, regardless of their responses. This approach was considered to minimize the impact of representation bias too. Only two individuals who were informed about the study did not participate. Furthermore, there is a risk of not being able to identify all potential participants during busy hours, which introduces potential bias as the study mainly included individuals who were able to visit the clinic during non-peak hours. This selection bias may result in the inclusion of participants who potentially have milder symptoms and may have different beliefs.

Due to pragmatic reasons, participants were recruited from two PHC located in distinct areas in Riyadh with different population compositions to enhance the sample’s variability. Although we recruited participants from the most culturally diverse city in Saudi Arabia (Riyadh), cultural representation of all Saudi Arabia population might still be limited. Despite these limitations, the study provides an in depth understanding of the beliefs about antibiotics held by members of public in Saudi Arabia.

## 5 Conclusion

The study identified several factors that can be targeted in future interventions to promote appropriate antibiotics use and reduce demand for antibiotics in URTIs. Some of these factors match those previously identified in the literature (e.g., symptom experiences, perceived accelerated recovery with antibiotics, and low concerns about antibiotics). Others were unique to the circumstances of this study, such as the belief that stronger and more powerful antibiotics are more harmful, and some misconceptions related to AMR. Most of these factors that can be operationalised using the NCF as a theoretical framework. The NCF has been extensively used to explain treatment beliefs related to chronic medication use but rarely in medication of short duration of use. Although understanding treatment beliefs for medication administered for short period of time might seems less essential. It is crucial to understand it in relation to antibiotics as treatment beliefs have an important role in shaping attitudes toward antibiotics use and thus influence the global threat of antimicrobial resistance. The study suggests that taking a tailored approach in designing these interventions that correspond to specific beliefs and needs of patients with active URTI symptoms is crucial. By addressing these specific beliefs, the intervention is more likely to be successful with patients and have a higher chance in promoting appropriate antibiotic use and positively impacting patient outcomes. Further investigation into the factors influencing patients’ inappropriate demand for antibiotics is crucial to effectively address this aspect of antibiotic prescribing.

## Data Availability

All relevant data presented in the study are included in the article/Supplementary Material. The raw datasets from this study are not readily available due to patient confidentiality and participant privacy. It is not possible to make patient data publicly available, as participants in the study did not provide consent to release their interviews/interview transcripts. Further inquiries can be directed to the corresponding author.
