# Supplementary material for: Understanding patient demand for and use of antibiotics for upper respiratory tract infection: A qualitative application of the Necessity-Concerns Framework in Saudi Arabia
Source: Front Pharmacol. 2024 Jun 19;15:1399698. doi: 10.3389/fphar.2024.1399698 (PMC11220495; doi:10.3389/fphar.2024.1399698)
Supplement: Supplementary file 1 [file DataSheet1.docx]

Supplementary Material

# supplement (1): Interview Discussion guide

## Introduction

Thank you for your time today and agreeing to take part in the interview

We would like to know more about your thoughts about antibiotics and antibiotic resistance, and how you feel about recent policy changes that banned selling antibiotics without prescription from pharmacies.

We will use a digital recorder to record our interviews because it’s difficult to write down everything and we don’t want to miss out anything you say and it will help us to listen to what you have to say with full attention. We would like to assure you that the recordings will stay confidential and will be listened to by one of the researchers anonymously to be transcribed.

We would like to confirm that anything you say will be anonymous. Your name and personal details will not be mentioned in any report. Your personal data will have a code number instead. Feel free to speak your mind, there is no right or wrong answers we are interested in your personal opinion.

This study is a part of a PhD project where we want to understand what are the public views about antibiotic use for cold/flu symptoms and about antibiotic resistance? We also want to explore how the public feels about the recent policy changes that banned selling antibiotics without prescription and how this would impact their access to healthcare.

The interview is expected to take 30minutes.

Do you have any question before we start?

## Domains

### Perception of consultation Attitude toward illness

- How do you manage cold/flu symptoms?
- **(Prompt: do you go to pharmacy for self-management or doctors)**
- How long after getting cold/flu symptoms do you wait before you see the doctor?
- What is your expectation from the consultation?
- **(Prompt: Get diagnosed, get a prescription)**
- Did the consultation meet your expectations or not and how?
- Did the physician make the decision about giving a prescription (antibiotics) or have you asked him/her for a prescription?
- **(Prompt: did you request it? Do you trust your doctor judgment about the need for antibiotics?)**
- What type of information were you provided regarding the use of antibiotics?
- **(Prompt: when to stop using them, reusing them, how to dispose them)**
- How satisfied are you with the consultation and why?
- **(Does getting antibiotics related to your satisfaction or not?)**

### Necessity of Antibiotics

- How do you feel about taking antibiotics?
- Do you think that you need antibiotics, why?
  - When making your decision whether you need antibiotics or not, what factors would make you feel you need antibiotics (ie encourage you from getting them)?
  - What would make you feel you did not need antibiotics (ie discourage you from getting them)

### Concerns about antibiotics

- Do you think antibiotics can be harmful? How?
- Do you have any concerns about using antibiotics if prescribed?
  - When making your decision whether you need antibiotics or not, what things would influence your concerns about antibiotics?
  - What would help increase / decrease these concerns?

### Knowledge about antibiotics and antimicrobial resistance (AMR)

- When do you think antibiotics should be prescribed if patient have upper respiratory tract infection symptoms?
- **(Prompt: type of infection bacterial or viral)**
- Some people think that frequently taking antibiotics can make it harder to treat other infections. What do you think about this?
- Have you heard about antimicrobial resistance (AMR) what do you know about it?
- **(Prompt: causes, spread, and deterioration)**
- What can you do to help control the spread of AMR, and how?

### Recent policy changes

In March 2018, almost 2 years ago, the Ministry of Health in Saudi Arabia had reinforced a policy that banned selling antibiotic without prescription. This mean that you can no longer go to the pharmacy and ask the pharmacist to dispense antibiotics. You will have to go to a doctor to get diagnosed and get prescription for the antibiotic if you need one.

- How do you feel about banning selling antibiotic without a prescription?
- How do you get access to antibiotics now if you need them?
- If your doctor does not prescribe you an antibiotic, what would you do?
- **(Prompt: will you go to another doctor? Or will you accept the decision)**
